# Tetanus

**Published:** 1894-10

**Authors:** Charles J. Montgomery

**Affiliations:** Augusta, Ga.


### TETANUS. *

BY CHARLES J. MONTGOMERY, M.D.,

AUGUSTA, GA.

Tetanus is defined as "a disease of the nervous system characterized by persistent tonic contraction of some or all of the voluntary muscles."† It is defined as a disease of the nervous system, "because it is through the medium of the nerves and spinal cord that its phenomena are manifested, and because the nervous system alone has as yet been found to present *post mortem* changes with sufficient constancy to be considered significant." I would suggest as a definition, one which seems to me more accurate and in accord with modern views, which is as follows:

Tetanus is an infectious disease, due to a specific micro-organism and usually of traumatic origin characterized by tonic contraction and convulsions of some or all of the voluntary muscles. Tetanus may be acute, subacute, or chronic, but these terms refer chiefly to the length of time of incubation, being of the acute variety when the incubation period is very short, from a few hours to a day or two; and of the subacute or chronic form, according as the onset of the disease has occurred five or six days or more after infection has

---

* Read before the Augusta Academy of Medicine. See page 470.
† Ashhurst.

taken place. These divisions are important from a prognostic standpoint, the prognosis being more grave the more acute the disease, both as regards the brevity of the incubation period, and the severity of the symptoms.

Tetanus is also divided into traumatic, idiopathic, puerperal and tetanus nascentium. I believe the latter three might be considered as subdivisions of the first, as the cause and symptoms of all are essentially the same. *Traumatic* tetanus is of course that form of the disease following a wound. *Idiopathic* is that variety in which the point of infection is not demonstrable, analogous in *this* respect to what was formerly called idiopathic erysipelas. In *puerperal tetanus* the traumatism, through which the poison gains access to the system, is probably in the genital tract, while *tetanus nascentium*, which according to Parrott partakes more of the nature of uremic eclampsia than of true tetanus, is regarded by Marion Sims and others as traumatic in origin, due to a displacement of one of the cranial bones, though it is quite probable that infection takes place at the division of the umbilical cord.

ETIOLOGY OF TETANUS.—The predisposing conditions may first be mentioned, such as, male sex, early adult life, negro race, improper hygienic surroundings, exposure to cold and wet and sudden changes of temperature from warm to cold. Now, having dwelt on the necessity of a wound as a prime factor in the production of the disease, I come to what may be called its special etiology. Here we find different theories: First, that it is due to an abnormal condition of an inflammatory nature, affecting a peripheral nerve, which condition extends by continuity of structure directly to the spinal cord; or else, that this inflammation of the termination of a centripetal nerve causes, by reflex action, a general spasm. Holmes, in his "System of Surgery," says: "I am really surprised that some persons still doubt that it is owing to a peculiar influence exerted on the circulation of the blood and the nutrition of the spinal cord and the medulla oblongata, by the irritation of a centripetal nerve, that tetanus arises from a traumatic lesion." This theory seems to be supported more or less by clinical experience; thus, after the removal of a foreign body which has been pressing on a nerve, decided improvement has followed. Occasionally, though rarely, section of the principal nerve terminating at the seat of injury has caused a distinct amelioration of the symptoms. Also, the disease has apparently been caused by "a splinter of bone sticking in the radical nerve, by a portion of whip embedded in the cubital nerve, by the application of caustic potash on the coraco-brachial nerve," and many other like instances of its apparent causation might be adduced.

The second view, which is the one in accord with more recent investigations, is that the disease is due to the product of some poison excreted by a living organism; and this leads me to speak of the tetanus bacillus. This bacillus, discovered almost simultaneously by Nicolaer and Rosenbach, is described as an anaërobic micro-organism with "a spore at one of its extremities which gives it the resemblance to a pin or drum-stick." The bacilli have been found active after exposure for an hour to moist heat at 176° F., but when this heat is raised to 212° F., and maintained at this temperature for five minutes, they are destroyed. Direct sunlight will destroy them in from fifteen to eighteen hours. This bacillus has been found in soil and in street dust, and in men suffering with tetanus; it has appeared "in the wound secretions, in the nerves leading from the seat of infection, and in the spinal cord." Four ptomaines of the bacillus tetani have been isolated by Brieger, which he calls, first tetanin, second tetanotoxin, third muriate of toxin, and fourth spasmotoxin. Any one of these injected subcutaneously in mice produces symptoms more or less characteristic of tetanus; but the third, that is muriate of toxin, in addition, "excites the salivary and lachrymal glands to increased functional activity."

SYMPTOMS.—The *symptoms* of tetanus may develop gradually or quite suddenly. If the former, they will usually be first manifested by a feeling of malaise and general discomfort, such as precede many other diseases and will in no wise be indicative of tetanus. Usually the first symptom which causes the nature of the disease to be suspected is "a feeling of stiffness, with pain on motion, affecting the muscles of the lower jaw and tongue, and those of the back of the neck." Sometimes, however, preceding this there may be pain or cramps in the affected limb. Usually dysphagia soon develops, and then comes the characteristic symptom, trismus, which gives the disease its popular name, lockjaw. Here the teeth of the upper and lower jaw may be clinched together, or there may be a sufficient space between them to allow liquids to be ingested. There is always a tendency to convulsions, usually more marked in the muscles of the back, and when these muscles are the ones chiefly affected in the convulsions, the patient is in a condition of opisthotonos. When the body is bent forward, this condition is know as emprosthotonos, while in pleurothotonos the convulsion bends the patient to one or the other side. However, while one group of muscles is more intensely convulsed, in reality all of the voluntary muscles, including the diaphragm, are usually more or less affected. Pain from the precordial region to the spine, said to be due to a spasm of the diaphragm, is considered a characteristic symptom of the disease, though it is not always present. A peculiar expression, known as the tetanic grin, may often be noted.

The fever is usually not high, the temperature being higher during a convulsion, and in fatal cases it may rise very high just before death, having been recorded as high as "112.5° F. at the time of death, and 113.5° F. a short time subsequently." There is usually no delirium, though rarely it is present in a low form. Death may take place during a convulsion from apnea, or later in the course of the disease from exhaustion.

PATHOLOGY.—The pathological conditions are neither constant nor characteristic, but there is found to be present a myelitis, affecting chiefly the posterior cornua of the spinal cord and the contiguous central gray matter, which "at times invades the related white columns." The disintegrated gray matter is found to be in a state of solution in some places, holding in suspension fragments of disintegrated tissue. Lockhart Clarke believes that "the lesions depend on the conjoint operation of injury of the peripheral nerves with hyperemia and a morbid state of the blood-vessels of the cord, and the resulting exudations and disintegrations." According to Wyeth, "at times no appreciable interference with the nutrition of the gray matter can be detected," though this must be extremely rare. The cervical portion of the cord is that seen to be most affected.

In the *diagnosis* of tetanus there are several conditions to be considered, the chief of which are, spinal meningitis, hydrophobia, strychnine poisoning, and hysteria. In *spinal meningitis* there is not the characteristic early trismus, and in the intervals of the spasms, the fixation of the muscles is largely voluntary, to prevent pain on motion. In *hydrophobia* the convulsions are clonic, not tonic, delirium is the rule, not the exception, as in tetanus, and there is an abhorrence of water and other liquids. In *strychnine poisoning* the diagnosis is usually determined by the history of the case, but when this is not attainable, we especially note that between the paroxysms there is complete relaxation, and no fixation of the jaw. *Hysteria* has been mistaken for tetanus, but it must be extremely rare, that a careful observer could make this mistake. The *prognosis* of tetanus is always grave, and more grave, the more acute the disease.

TREATMENT.—This should be general and local. In this, as in all other dangerous diseases, attention to details and attention to treatment other than by drugs is of the utmost importance. Under general treatment I will first mention hygiene. The patient should be kept in a moderately dark, warm, dry room; he should be especially protected from draughts, sudden noises, sudden exposure to light, etc. Hot applications, both wet and dry, should be made to the neck and spine; they are doubtless much better than cold applications, which are apt to be more or less depressing. He should be subjected to massage of the spine, jaws and limbs, especially with hot cocoa-butter or hot oil. I have found this adjunct to other treatment of decided benefit, and believe it is of service in three ways: First, the heat and rubbing acts as a counter-irritant to the spine; second, by its local effect on the muscles; third, enough is probably absorbed to aid in nourishing the exhausted system. The patient should receive concentrated nourishment, such as milk and whisky, at frequent intervals, if he can swallow; otherwise, rectal alimentation, and rectal or hypodermic medication, or both, must be practiced. His bowels should be moved at the outset by a laxative, and subsequently every two or three days, an enemata may be given. I now come to drugs. A score or more have been recommended and used, with varying degrees of success and failure. Of these, those of most benefit seem to be chloral and bromide of potassium in large doses, opium, calabar bean, curare, etc.; though recovery has followed the administration of quinine, and also of gelsemium, not to speak of many others which have been suggested. Bacelli's treatment is by the hypodermic injection of carbolic acid, and a case is reported in which carbolic acid injections, one-sixth grain every two hours, effected almost immediate relief, ending in recovery, after chloral, bromide of potassium and hypodermic injections of quinine had all failed. During a convulsion, chloroform might be given by inhalation, or if the chest be immovable, nitrite of amyl hypodermically, while it would rarely be practicable then to administer chloral and bromide by the rectum.

Before leaving the subject of the general treatment of tetanus, I wish to say a word on a few recent investigations which have been made on the action of blood-serum on the poison of tetanus, and the results of treatment suggested thereby. It has been found by experiment that by repeated hypodermic injections of small doses of the poison of tetanus into an animal susceptible to the disease, he will be rendered immune, and can then "tolerate with perfect impunity many thousand times the amount of the poison," which would be fatal to the unprotected animal. Also that the serum of the blood of this immunized animal has the power of conferring immunity on another animal. Another interesting point in this connection is that, given an animal such as the fowl, which is not susceptible to tetanus, its blood-serum has no antitoxic power, but may be rendered antitoxic after a time, by a large injection into this non-susceptible animal of the tetanus poison. An animal once rendered immune remains so for a long time, if not indefinitely.

After tetanus in an animal has once developed, injections of antitoxic serum may or may not be followed by recovery, but cannot be relied on in effecting a cure.

The treatment of tetanus in man by injections of antitoxic serum has been tried by different observers, notably by Roux and Vaillard, who, out of seven cases thus treated, reported five deaths, or a mortality of about seventy-one and one-half per cent. Thus, while indications point to the probability that man may be rendered immune to tetanus, yet when the disease is already developed, antitoxic serum seems to have but little effect, though the question is still *sub judice*, and as antitoxic serum has been shown to be harmless even in very large doses, it might be used in conjunction with other measures.

The *local* treatment of tetanus is important. The wound must be thoroughly examined, and if any foreign bodies are present, they should be removed and the wound disinfected and dressed antiseptically. If the wound has healed, the scar should be excised and the resulting wound dressed with an anodyne, such as laudanum combined with iodoform, the latter having been found to be one of the most active disinfectants on the virus of tetanus, but can only do good on the surface of the wound, and when the disease is already developed cannot arrest its progress, so the earlier it is applied the more beneficial will be the results. A dressing to be recommended for the wound made by the excision of the scar is one of iodoform, covered with iodoform gauze moistened with laudanum, and this covered with rubber tissue, cotton, and a roller bandage. Before applying the dressing, however, some disinfecting solution or cauterizing agent should be applied.

If the wound be not excised, it is well to make a V-shaped or U-shaped deep incision, separating the nerve connection between the wound and spinal cord. So far as amputation in tetanus is concerned, statistics seem to be against it unless otherwise indicated.

---

### AN EXAMINATION OF THE ABDOMEN IN THE GENU-PECTORAL POSITION.

Plique recently, in a communication to the Paris Academy of Medicine, spoke of the advantages of this position. The thighs should be slightly flexed, and if this be done the author considers it the best position to obtain complete relaxation of the abdominal walls. While useless in the case of large tumors, it is very valuable for the examination of the hypochondriac regions, the flanks, the iliac fossæ, the pelvis, and in the search for small tumors. It is contraindicated in cases of dyspnœa and advanced pregnancy.

